# Darkfield Adapter for Whole Slide Imaging: Adapting a Darkfield Internal Reflection Illumination System to Extend WSI Applications

**DOI:** 10.1371/journal.pone.0058344

**Published:** 2013-03-08

**Authors:** Yoshihiro Kawano, Christopher Higgins, Yasuhito Yamamoto, Julie Nyhus, Amy Bernard, Hong-Wei Dong, Harvey J. Karten, Tobias Schilling

**Affiliations:** 1 Olympus America, Center Valley, Pennsylvania, United States of America; 2 Olympus Corporation, Hachioji, Tokyo, Japan; 3 Allen Institute for Brain Science, Seattle, Washington, United States of America; 4 David Geffen School of Medicine, University of California Los Angeles, Los Angeles, California, United States of America; 5 Department of Neurosciences, School of Medicine, University of California San Diego, La Jolla, California, United States of America; 6 Olympus Soft Imaging Solutions GmbH, Münster, Germany; Emory University, United States of America

## Abstract

We present a new method for whole slide darkfield imaging. Whole Slide Imaging (WSI), also sometimes called virtual slide or virtual microscopy technology, produces images that simultaneously provide high resolution and a wide field of observation that can encompass the entire section, extending far beyond any single field of view. For example, a brain slice can be imaged so that both overall morphology and individual neuronal detail can be seen. We extended the capabilities of traditional whole slide systems and developed a prototype system for darkfield internal reflection illumination (DIRI). Our darkfield system uses an ultra-thin light-emitting diode (LED) light source to illuminate slide specimens from the edge of the slide. We used a new type of side illumination, a variation on the internal reflection method, to illuminate the specimen and create a darkfield image. This system has four main advantages over traditional darkfield: (1) no oil condenser is required for high resolution imaging (2) there is less scatter from dust and dirt on the slide specimen (3) there is less halo, providing a more natural darkfield contrast image, and (4) the motorized system produces darkfield, brightfield and fluorescence images. The WSI method sometimes allows us to image using fewer stains. For instance, diaminobenzidine (DAB) and fluorescent staining are helpful tools for observing protein localization and volume in tissues. However, these methods usually require counter-staining in order to visualize tissue structure, limiting the accuracy of localization of labeled cells within the complex multiple regions of typical neurohistological preparations. Darkfield imaging works on the basis of light scattering from refractive index mismatches in the sample. It is a label-free method of producing contrast in a sample. We propose that adapting darkfield imaging to WSI is very useful, particularly when researchers require additional structural information without the use of further staining.

## Introduction

### 1. History of virtual slide imaging

Biomedical researchers have been using optical microscopes to investigate tissue and cells for more than 400 years. Optical microscopy is a useful tool for observing fine tissue detail and cellular structure. However, there are notable limitations. When a specimen is observed using a lower-magnification and/or lower numerical-aperture (NA) objective lens, the resulting widefield image is lower in resolution. Using higher-magnification objectives with greater NAs (such as a typical 20×, NA 0.75 objective lens), the resolution is improved but the image’s field of view is markedly restricted. This major limitation is especially obvious in the field of neuroscience. For instance, a detailed analysis of the cortex requires direct sampling and comparison of vast areas of tissue. This requires images as large as 200×250 mm, with a resolution of at least 0.5 micrometers/pixel in order to detect relevant cellular properties.

In the past, scientists such as Korbinian Brodmann and Cécile and Oskar Vogt used high-resolution emulsion films designed for astronomy to attempt to capture the fine detail essential to their cytoarchitectural analyses. However, the larger field of view in their photographs resulted in lower optical resolution consequent to the limitations of the NA. In order to compensate for the optical and emulsion deficiencies of their photographic apparatus, they attempted to capture the complexity of the neural tissue by using a microscope drawing tube to sketch a high-resolution image in widefield [1]. But sketching multiple fields of microscope images proved to be difficult and time consuming, and it often introduced errors in the system, including both major sampling bias and inaccuracies arising from the burdensome procedure of attempting to represent cell density and cell size accurately.

Technological advances have led to the creation of robotic microscopes. In addition, image-stitching technologies, cost-effective data storage systems and local- and wide-area networks (LAN and WAN) are now widely available. Pathology is one area that has compelling application and utility for WSI and image servers [2]–[4]. In the fields of research pathology, education and nonclinical environments, widefield microscope images today often are acquired using whole-slide or virtual-slide-scanning microscopes [5]–[8].

### 2. History of Brain Mapping

Neuroscientists have recognized several advantages that WSI imaging presents when compared with conventional optical microscopy. Remote viewing, data sharing, and various forms of data mining are just some of these advantages [9], [10]. The large field of view is another advantage: the brain has complicated and inter-tangled masses of neural networks and structures that extend beyond the field of view of almost any conventional microscope objective lens.

Virtual slide technology provides the high-resolution imaging required for widefield observation. It also provides an excellent tool for observing brain neural systems and structures that are widely distributed over many serial sections. One of the most powerful applications of WSI is the ability to scan a complete set of serial sections within a brief period of time. This advantage greatly simplifies the task of analysis and allows researchers to share the research results with colleagues thousands of miles away. WSI has also enabled neuroscientists to provide internet-enabled high-resolution brain maps and atlases to display neural connections and image protein expression [9]–[12].

### 3. Virtual Microscopy Observation Methods

A conventional optical microscope provides numerous specimen observation methods such as transmitted light, brightfield, fluorescence, differential interference contrast (DIC), phase contrast, polarized light and darkfield imaging. Darkfield microscopy, in particular, has been widely used to detect silver grains consequent to autoradiography (ARG), gene expression and immunohistochemically-labeled cell bodies in sliced brain tissue for many years [13]. It also has been used to observe and analyze complex pattern of axonal projections labeled with anterograde tracers, providing enhanced signal-to-noise-ratios (SNR). Darkfield images can reveal exquisitely detailed morphologies, such as axonal boutons and varicosities [14]–[16]. However, although WSI systems are commercially available from various companies and may be used to collect high-resolution widefield microscope images, current virtual slide systems are mostly limited to brightfield and fluorescence imaging; they do not provide high magnification darkfield imaging. We sought to develop a method of combining darkfield imaging with WSI technology to overcome these limitations.

## Materials and Methods

### 1. Observation Methods Explained: Darkfield

A typical WSI specimen slide consists of three parts: a slide glass, the tissue or cells together with mounting media, and the cover glass. Brightfield imaging is the most commonly used light microscope illumination method. It uses transmitted illumination light, which travels from one side of the specimen through the specimen to a detector on the other side. As a result, the specimen is displayed on a bright background. In darkfield microscopy, illumination light enters the specimen at an oblique angle and is blocked from the detector. Therefore, the darkfield image background is dark and the specimen appears bright. If there is a mismatch between the refractive index of the specimen and the mounting media, the specimen will scatter or refract light. This illumination method provides enhanced detection sensitivity and contrast for specimens that are not imaged well using brightfield illumination. Using darkfield imaging, researchers in a wide variety of fields report detecting very small refractive index mismatches to elucidate specimen structural and surface attributes in detail [17]–[23].

There are several types of darkfield illumination. The first, transmitted darkfield illumination, is widely used for biological specimen observation. Typically, a transmitted darkfield illuminator uses a halogen bulb with either a dedicated substage darkfield condenser or a darkfield central stop in a standard brightfield condenser. Central axial illumination is blocked by the darkfield central stop and the peripheral illumination obliquely illuminates the specimen from below. Thus, only forward-scattered or refracted light from the specimen enters the objective lens. This light creates the darkfield images [17], [18].

A second type is reflected darkfield illumination. This method is commonly used to visualize metallurgical specimens and is not commonly use for biological specimens. Reflected darkfield illumination employs a vertical illuminator mounted above the objective lens. Only peripheral rays of light from the illuminator reach the deflecting mirror above the objective lens. The peripheral rays are deflected downward and around the periphery of a specially modified darkfield objective lens with a circum-objective condenser in the form of a doughnut. Only backward-scattered or reflected light from the specimen enters the objective lens creating the darkfield image [17], [18]. This method is of limited value with typical histological slides, as the light reflects off the coverslip.

A third type of darkfield illumination is side-illuminated darkfield. This method eliminates light sources from both top and bottom. A fiber-optic bundle is mounted horizontally at the edge of the slide so that light strikes the specimen from the side [17]. Only side-scattered or reflected light from the specimen enters the objective lens. The surrounding regions of the microscopic field present as a darkfield image. We refer to this as darkfield internal reflection imaging (DIRI).

### 2. Theory of Darkfield Internal Reflection Imaging (DIRI)

**Figure 1:**
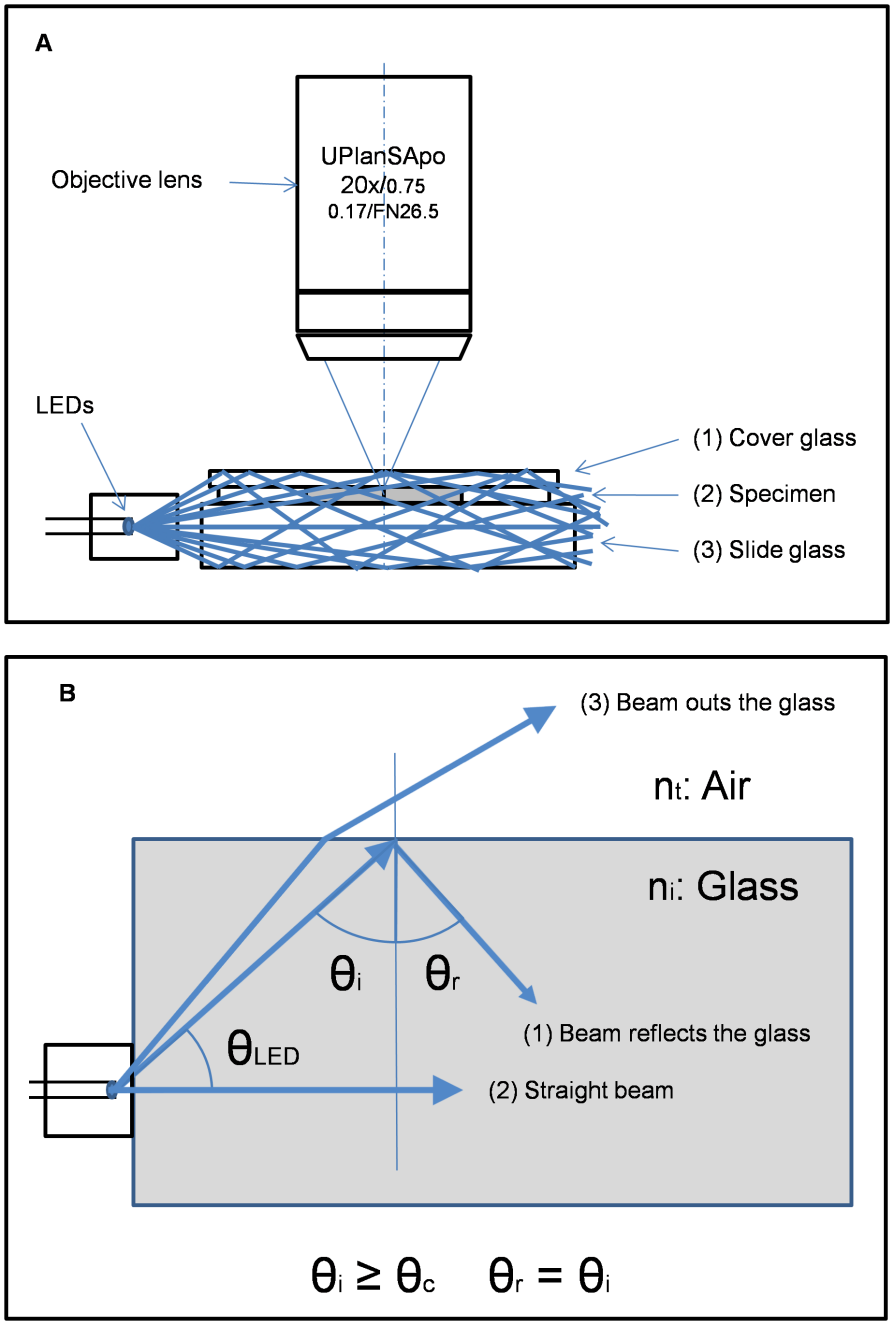
Mechanism of darkfield internal reflection illumination. (1A) Light hits the surface where air (n_t_) meets glass (n_i_). If light enters the surface, θ_i_ is more than the critical angle necessary to cause reflection from the surface. (1B) Light path within the sample when the angle of light is greater than the critical angle.



Snell’s Law, a formula describing how light travels though a boundary between two media with different refractive indices, helps elucidate the mechanism for DIRI-type system. Typically, the refractive index n_i_ of the cover glass, tissue or cells with mounting media and glass slide is 1.4 to 1.5. In contrast to this, the refractive index for air n_t_ is 1. Internal reflection (Figure 1A) occurs when n_i_>n_t_ and the incident beam angle θ_i_ is equal to or greater than θ_c_, the so-called critical angle [21]. If the incident beam angle is greater than critical angle, light reflects off of the surface between n_t_ and n_i_ (Figure 1B).

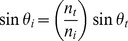



To find the critical angle if θ_t_ is 90 degrees then sin θ_t_ is 1.
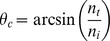



### 3. Experimental Setup for DIRI Using a WSI System

**Figure 2:**
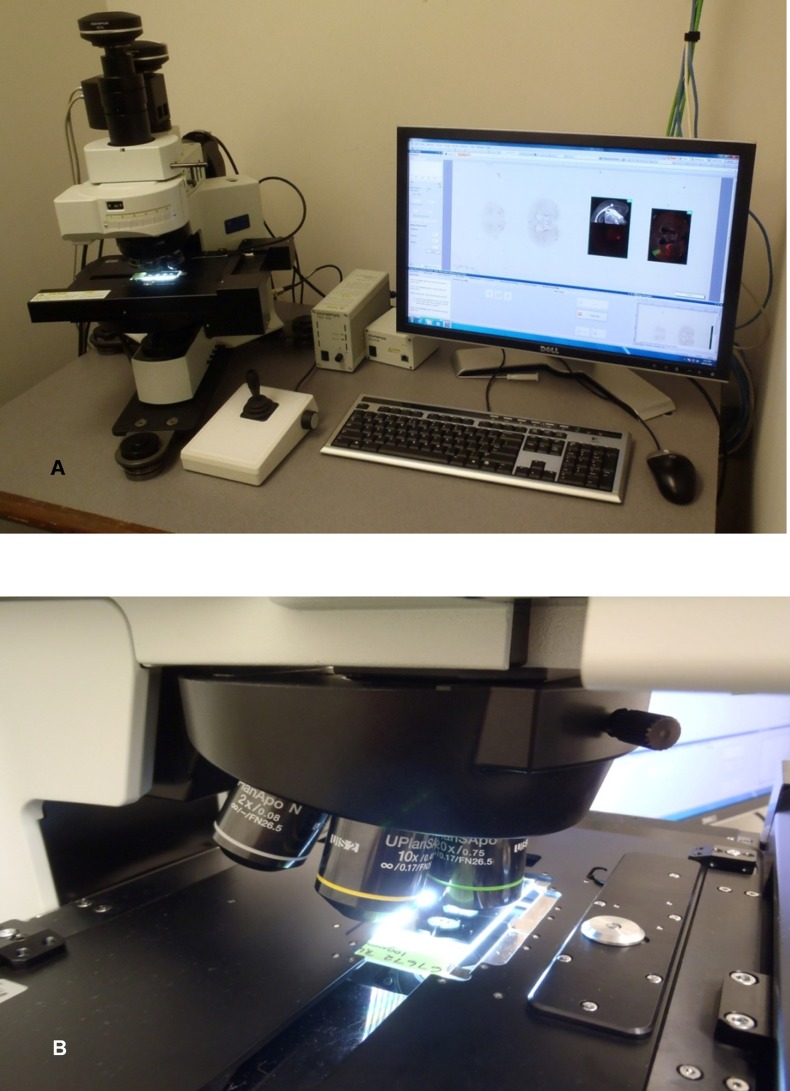
WSI system setup. (2A) The whole-slide imaging (WSI) system is configured on a microscope with motorized Z-axis focus capability, a motorized X-Y stage, the darkfield illuminator with stage insert plate, a mercury light bulb for fluorescence illumination (not visible here), a transmitted illuminator for the halogen light bulb (not visible here), and an XC10 CCD color camera (front camera) and XM10 CCD camera (back camera) to acquire transmitted brightfield and darkfield images. The WSI system is controlled by VS-ASW v2.5 software. (2B) Light scatter from a brain slice specimen. A brain slice is mounted on a 1×3-inch microscope glass slide and placed on the stage. A stage insert plate with multiple LEDs is mounted on the stage. The LEDs illuminate the entire microscope slide. The brain and mounting media have refractive index mismatches that cause the specimen to scatter light.

We used the VS120 WSI scanning system (Olympus America Inc., Center Valley, PA), which was configured for acquiring and storing widefield microscopic images (Figure 2A). The WSI system setup included a microscope imaging system with a camera, objective lens, motorized focus system and motorized X-Y stage. The system was equipped with a standard substage darkfield condenser, a transmitted brightfield illuminator, a fluorescence illuminator and the DIRI system described herein (Figure 2B). This configuration allowed the fully automated acquisition of multiple specimen images utilizing all four illuminators.

The operator used software to configure the acquisition parameters, selecting the magnification (objective lens) and observation method (e.g. transmitted brightfield, fluorescence or darkfield); the system used the input to set the stage and focus positions and acquire images automatically.

Prior to collecting high-resolution WSI images, we captured macro images at 2× magnification in transmitted brightfield or fluorescence mode. These low-resolution images provided an overview of the specimen for later navigation and high-resolution image capture. The macro image function enabled easier set-up in particular regions of interest.

**Figure 3:**
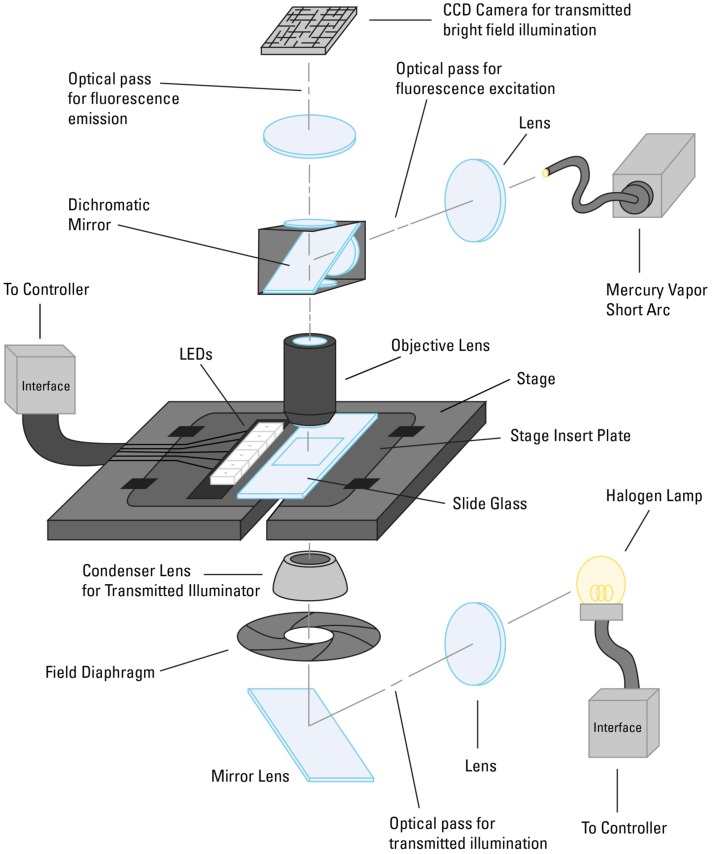
Schematic drawing of an experimental WSI system with DIRI. The DIRI is incorporated onto the WSI system’s motorized stage. LEDs illuminate the slide glass from the side, and the specimen scatters the illumination light. The scattered light is then collected by the objective lens above the stage. The dichromatic mirror on the motorized turret can be removed from the optical path when acquiring darkfield images. Above the dichromatic mirror is a tube lens that focuses the specimen image on the imaging device; a CCD camera captures the image.

For the acquisition of DIRI images, we developed a specialized illuminator (Figure 3). This illuminator is a variation on the typical side-illumination system, which is designed with a fiber-optic bundle integrated into the edge of the slide holder to introduce halogen light to the specimen; the traditional configuration is called a Hausmann’s darkfield illuminator [17] and is employed, for instance, in the commercially available Darklite Illuminator (Micro Video Instruments, Inc. Avon, MA). However, due to the constraints imposed by the use of a motorized stage with illuminator, the fiber bundle is rigid and can impede stage movement. We therefore chose LED illumination in place of the more-often-used rigid fiber bundle with halogen light illumination.

**Figure 4:**
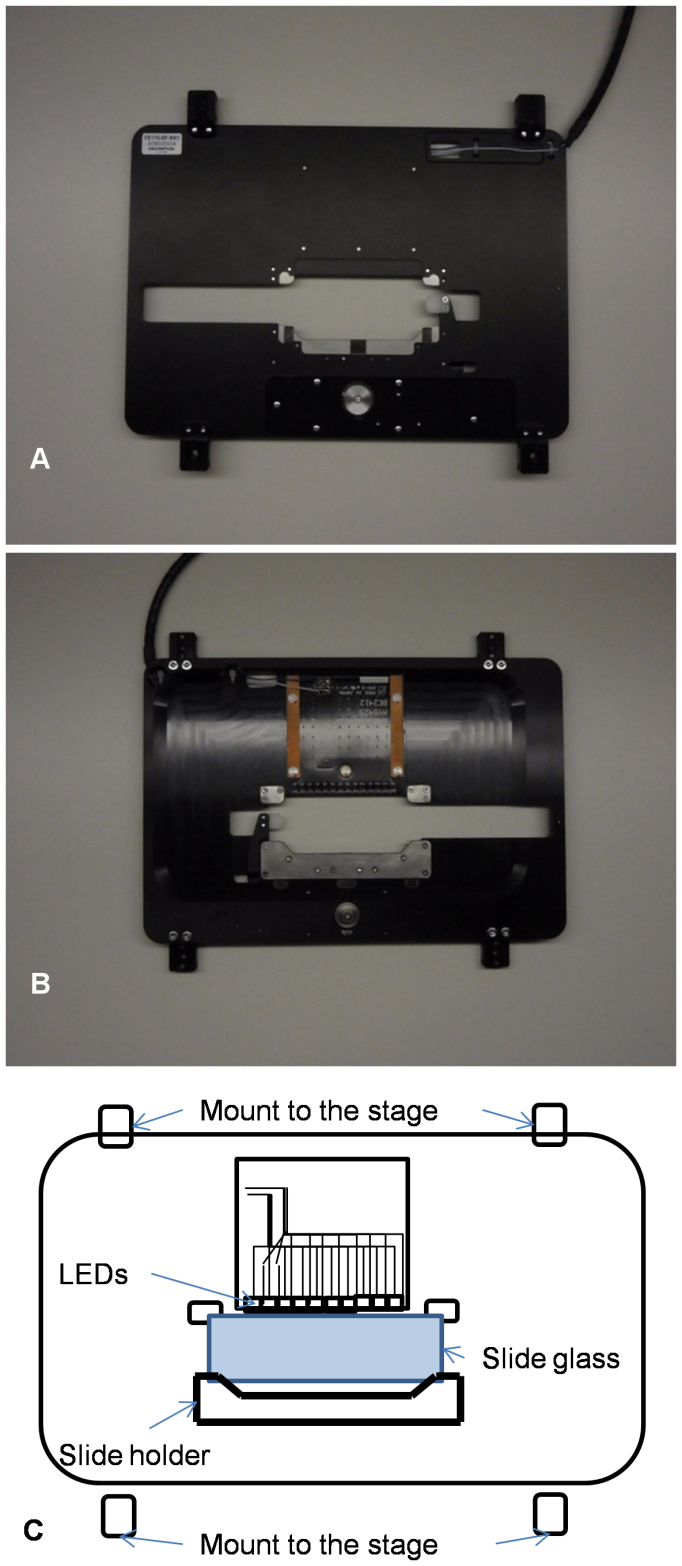
WSI with darkfield illuminator. (4A) Top view of the stage insert plate used with the darkfield illuminator. The plate has four black extension tabs that mount to the stage using screws. The microscope slide glass can be placed at the center of the stage insert plate. The round silver part is used to fasten the 1×3-inch microscope slide to the plate. (4B) Reverse views of the stage insert plate. The microscope slide glass is placed at the center. The LEDs are mounted on this side of the stage and illuminate this side of the slide glass. (4C) Schematic drawing of the WSI system with the darkfield illuminator. The 1×3-inch microscope slide glass is placed at the center of the stage insert plate. The four tabs are used to affix the plate to the stage.

Our darkfield illuminator utilized an LED light source (Figure 4A and B). To provide enough light intensity we configured multiple LEDs into a stage insert plate in a horizontal array (Figure 4C) that illuminated the specimen from many different angles simultaneously. The LED selected was a commercially available unit, the CL-435F (Citizen Electronics Co., Japan). This ultra-thin LED, originally designed to be incorporated into a mobile phone’s back panel illuminator, is just 0.4 mm thick.

The LEDs were equipped with clear plastic molded covers that provided a light guide function; the light sources illuminated specimen slides from edge to edge. The user could switch the illumination on and off via software during image acquisition.

According to the LED manufacturer, each LED’s maximum luminous intensity is 1380 mcd. The LED light’s angular distribution of 50% of the light intensity is plus/minus approximately 60-degree angulation. This means θ_ LED_ is about 60 degrees (see Figure 1B).

A light intensity control was added to the system, allowing the operator to control the light intensity and vary exposure times. We used an Illuminance Meter T-1 (Minolta Camera Co., Ltd., Japan) to measure light intensity with the light detector 3 mm from the LED array. The illuminator’s measurement result of maximum intensity is 3740 lux.

The slide glass and cover glass refractive indices are each about 1.5. Using this number, we calculated that the critical angle θc is 41.8 degrees. Transferring the critical angle θc 41.8 degrees to the LED illumination angle, the required LED illumination angle θ_ LED_ is less than 48.2 degrees. Thus, the LED illumination beam angle θ_ LED_ required to produce DIRI illumination is less than 48.2 degrees (see Figure 1B (1)).

Most of the LED illumination light beam θi is greater than the critical angle θc. These beams strike the surface where the glass meets air. This light beam reflects multiple times between the top glass surface and bottom glass surface, illuminating the entire specimen (Figure 1A). For LED illumination at the angle around zero degrees, the light beam goes though the slide glass and illuminates the specimen directly (see Figure 1B (2)). DIRI uses both of these mechanisms to illuminate the specimen. The LED illumination angle θ_ LED_ more than 48.2 degree is not used for DIRI because the illumination light is transmitted at the surface between air and glass (see Figure 1B (3)).

One unique feature of this experimental system was the ease with which we were able to acquire high-resolution images of the specimen using fluorescence, transmitted brightfield and darkfield at the same specimen location. To scan using brightfield, darkfield or fluorescence, the user selected a Scan button in the software, chose the type of scan, and then the system adjusted all settings automatically, including applying the appropriate dedicated background correction in real time during acquisition. The user could press the Scan button again to collect additional scans using the other observation methods. The system also provided seamless stitching of whole slide images for the collection of data that extended beyond any single field of view.

## Results

### 1. Imaging of DAB-stained Specimens Using DIRI

**Figure 5:**
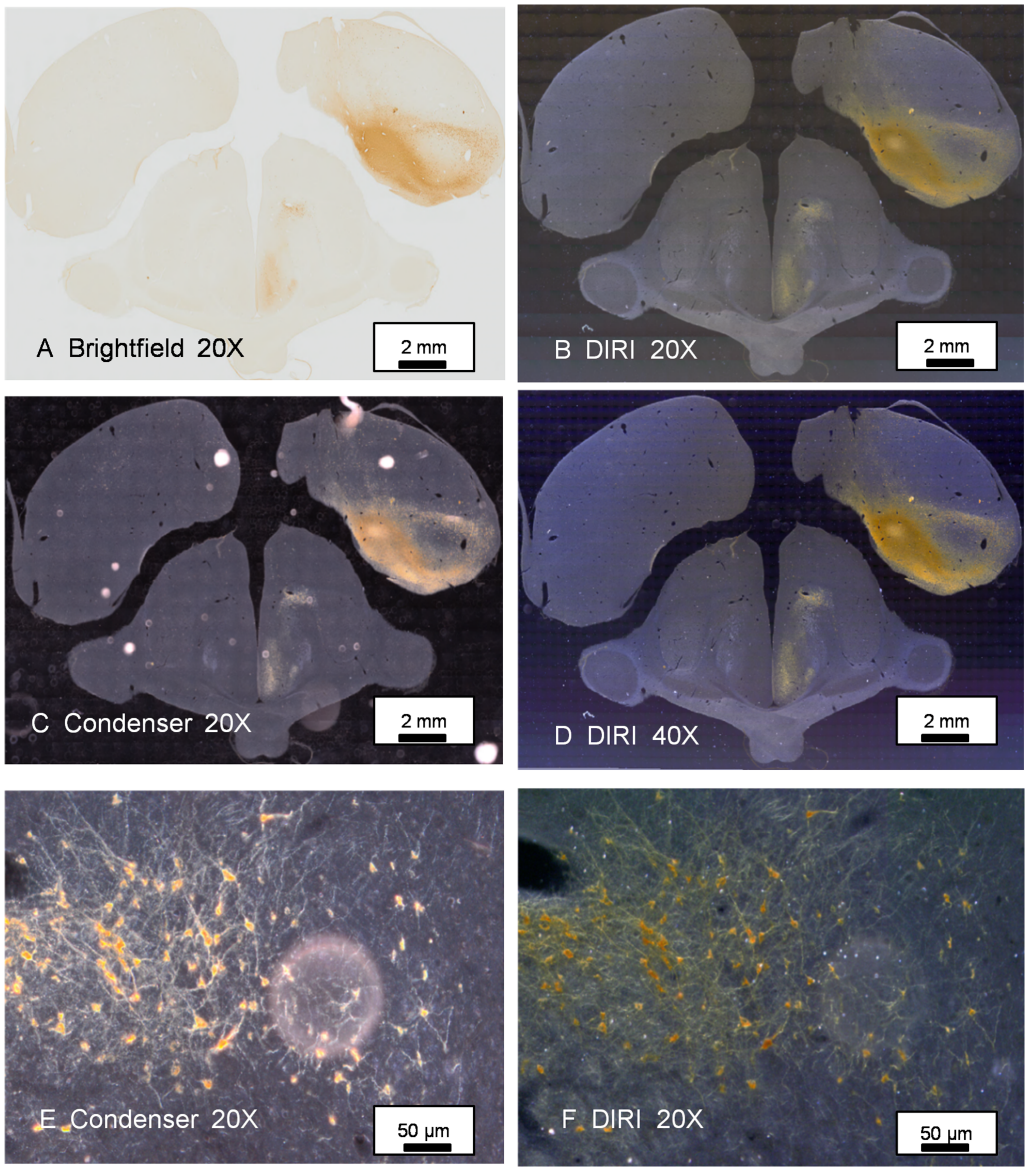
Comparisons of a variety of imaging methods used to depict DAB specimen showing anterograde and retrograde transport of Cholera Toxin B tracer in a chick brain with an injection of tracer in the medial Arcopallium (amygdala). Darkfield illumination with either a traditional darkfield substage condenser or with the edge-illumination method described herein readily demonstrates the presence of fine unmyelinated axons and dense terminal fields in the hypothalamus. (5A) DAB-stained brain section, 30 um thick, mounted on a 1×3-inch microscope slide glass and with a #1 coverslip. This WSI image was scanned in brightfield using a 20×, NA 0.75 objective lens. (5B) The same specimen was scanned using WSI with DIRI system and using the same 20× objective lens. (5C) WSI image with traditional darkfield substage condenser using 20× objective lens with motorized condenser lens (U-UCDB) with darkfield annulus (U-DFA). Light source is a halogen lamp. Dirt on the surface of the slide produces a number of distracting bright objects in the resulting image. (5D) WSI image with DIRI system using a 40×, NA 0.95 objective lens. Although a traditional motorized substage condenser and darkfield annulus are not compatible for use with an NA 0.95 objective lens, side-illuminated darkfield provides a clear, crisp darkfield image. (5E) WSI with traditional darkfield substage condenser and illuminator. Despite careful efforts to clean the slide, the specimen has some dust on the surface of the coverslip. The out-of-focus dirt obscures part of the image. (5F) WSI image of the same specimen shown in Figure 5E, captured using with DIRI system. The out-of-focus dirt on the coverslip, though still evident, is less intrusive.

DAB-stained brain sections were imaged with a 20× objective, using brightfield mode (Figure 5A), darkfield mode using DIRI (Figure 5B) and a traditional darkfield substage condenser, which was aligned and focused to conform to principles of Koehler illumination (Figure 5C). DIRI also was used with a 40× objective (Figure 5D). We did not collect any images with the substage darkfield condenser when using the 40× objective, as the severe mismatch of the lens NA and the substage condenser resulted in grossly inadequate darkfield images, with excessive glare, loss of contrast and degradation of the darkfield image.

A major shortcoming in the application of traditional darkfield imaging using a substage darkfield condenser has been the deterioration of the image due to dust (or artifacts) on either the top or bottom surface of the slide. To compare the DIRI images, with traditional darkfield, we examined the same specimens with dust on the top of the cover slip, using a darkfield substage condenser with a 20× objective lens (Figure 5E). The same specimen was also viewed with the side-illuminated darkfield. (Figure 5F). The out-of-focus dirt was less intrusive in the final image when captured using side-illuminated darkfield imaging.

Image acquisition conditions were as follows: Acquisition of one brain section (14.5 mm×9.7 mm) required 442 of the 20× images to build a single whole slide image (5A, B and C). This required 5 minutes, 21 seconds total acquisition time. Using a 40× objective (14.4 mm×9.6 mm) we needed 1734 images to produce a single whole slide image (5D). Acquisition at 40× required 21 minutes, 30 seconds.

**Figure 6:**
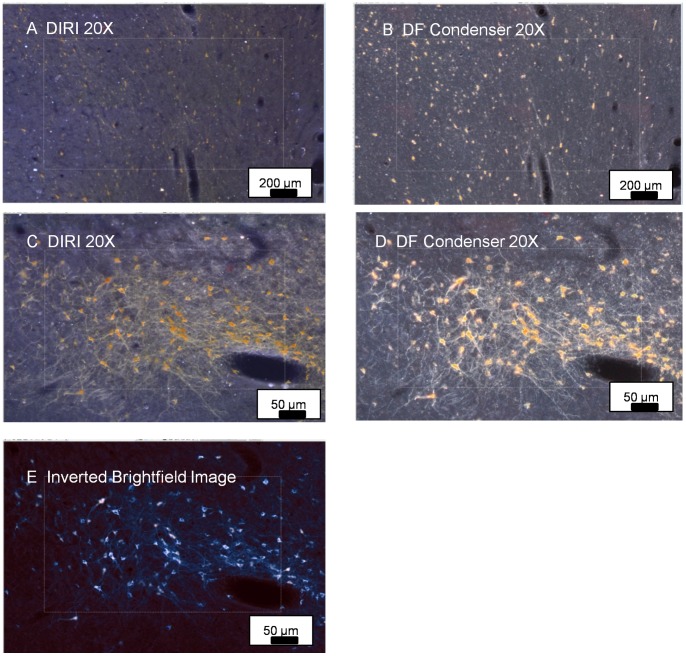
Comparison of matched pairs of WSI images using DIRI side illuminator and traditional darkfield substage condenser. Three WSI images using DIRI system (6A, 6C) are compared with traditional darkfield substage condenser images (6B, 6D). Each pair of photos (6A–B and 6C–D) depict the same region of a slide. The images using the DIRI system initially do not appear as robust as those taken with a darkfield substage condenser. Further examination of the images reveals that the images obtained with the DIRI system are more accurate representations of the cells and their processes. The substage darkfield condenser typically results in excessive light scatter. Differences in brightness and color of the individual cells with the DIRI system vs. the substage darkfield condenser are due in part to the difference in brightness of the illuminators and the spectral features of the illumination sources. (6E) shows the same region depicted in 6C–D, but is based upon a brightfield image that was then inverted to provide a “pseudo-darkfield” effect. Either darkfield method provides much better detail of the microscopic field than the inverted image in 6E.

To test the possibility that a simple inversion of the brightfield image (Figure 6E, F), using standard image processing software, might provide a satisfactory image of the fine detail evident following darkfield acquisition, we created an inverted brightfield image (Figure 6G). Although the cells were now shown in higher contrast and with a higher SNR than in the brightfield image, the finer detail of neuronal processes was no longer evident in the inverted image.

Using the 20× objective, we then compared exposure times for capturing an image of the DAB-stained brain slice specimen using the transmitted-condenser-darkfield illuminator and then using DIRI. Transmitted darkfield required an exposure time of 5 msec. DIRI required an exposure time of 700 msec. The exposure time for DIRI was longer than that of the transmitted-condenser-darkfield illuminator. Transmitted darkfield also provided a higher-contrast image. The disparity in exposure times was largely attributable to the overall lower intensity of illumination provided by the edge-illuminating LEDs, compared to the high-intensity halogen light source used with the substage darkfield condenser.

In addition, substage condenser illumination exaggerated staining due to the recruitment of substantial quantities of out-of-focus information. The DIRI side-illumination system did not require precise alignment of a substage condenser, worked well with objectives at all magnifications, and was relatively immune to small particles on the surface of the slide or coverslip.

Using darkfield illumination with a traditional substage condenser (Figure 6B, D and F) produced images with very high signal/noise ratios, with prominent halos around somata of neurons, dendrites and axons. Though the resulting images provide high contrast of the cells and processes, cells and processes appear to be much larger than their dimensions using traditional methods of imaging with transmitted light and differential interference contrast. However, DIRI (Figure 6A, C and E) produces much less halo using the side-scatted light illumination. In the resulting side-scatted light illumination DIRI images, neuronal somata appear as more accurate representations of the true sizes of the cells and processes.

### 2. Superimposing Images Using Fluorescence and DIRI

Fluorescence imaging is another modality used to observe proteins and gene distributions in tissue. Scientists often use immune-labeled fluorescence markers, genetically engineered fluorescent proteins and fluorescence *in-situ* hybridization (FISH) to observe specific proteins or genes using a fluorescence microscope. To ensure accurate analysis of the distribution of genes or proteins in the tissue, these techniques usually require counter-stains to visualize the corresponding cytoarchitecture of the specimens. However, counter-staining may be problematic if multiple proteins or genes are involved. Most fluorescence microscopes have three-to-five fluorescence channels and scientists usually prefer not to lose one fluorescence channel to counter-staining to observe cytoarchitecture information because that reduces the number of fluorescence channels available for investigating proteins or genes. DIRI provides a potential solution by providing cytoarchitecture information based on refractive index mismatches without reducing the number of channels available for fluorescence visualization (DIRI is not for visualizing a fluorescent molecule image).

**Figure 7:**
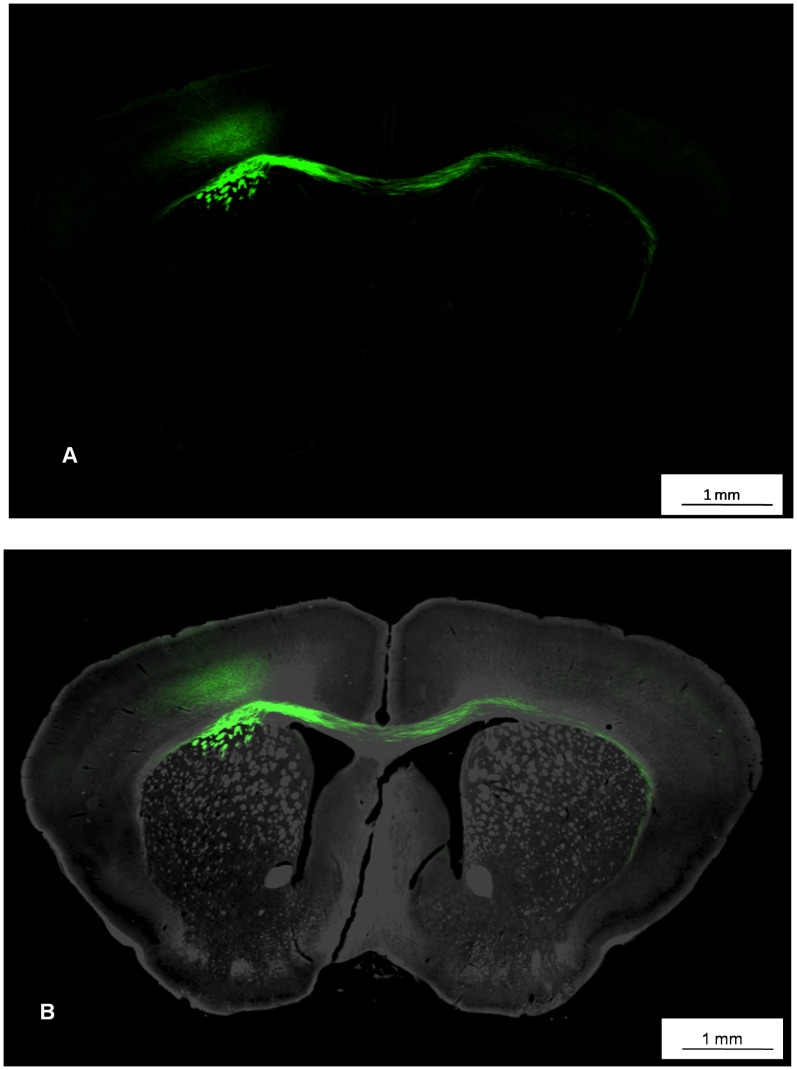
Visualization of the anatomy of a coronal mouse brain slice with fluorescent cell tracer. (7A) Fluorescent images are captured using a 10× objective lens. (7B) The fluorescent image is superimposed on a darkfield DIRI image. The superimposed image provides substantially more detail and anatomical context; the darkfield image depicts neuronal cell bodies and fibers while fluorescent label provides visualization of selected axonal projections. Acquisition time was 40 seconds for a 10× fluorescent image and 5 minutes for a 20× darkfield image.

**Figure 8:**
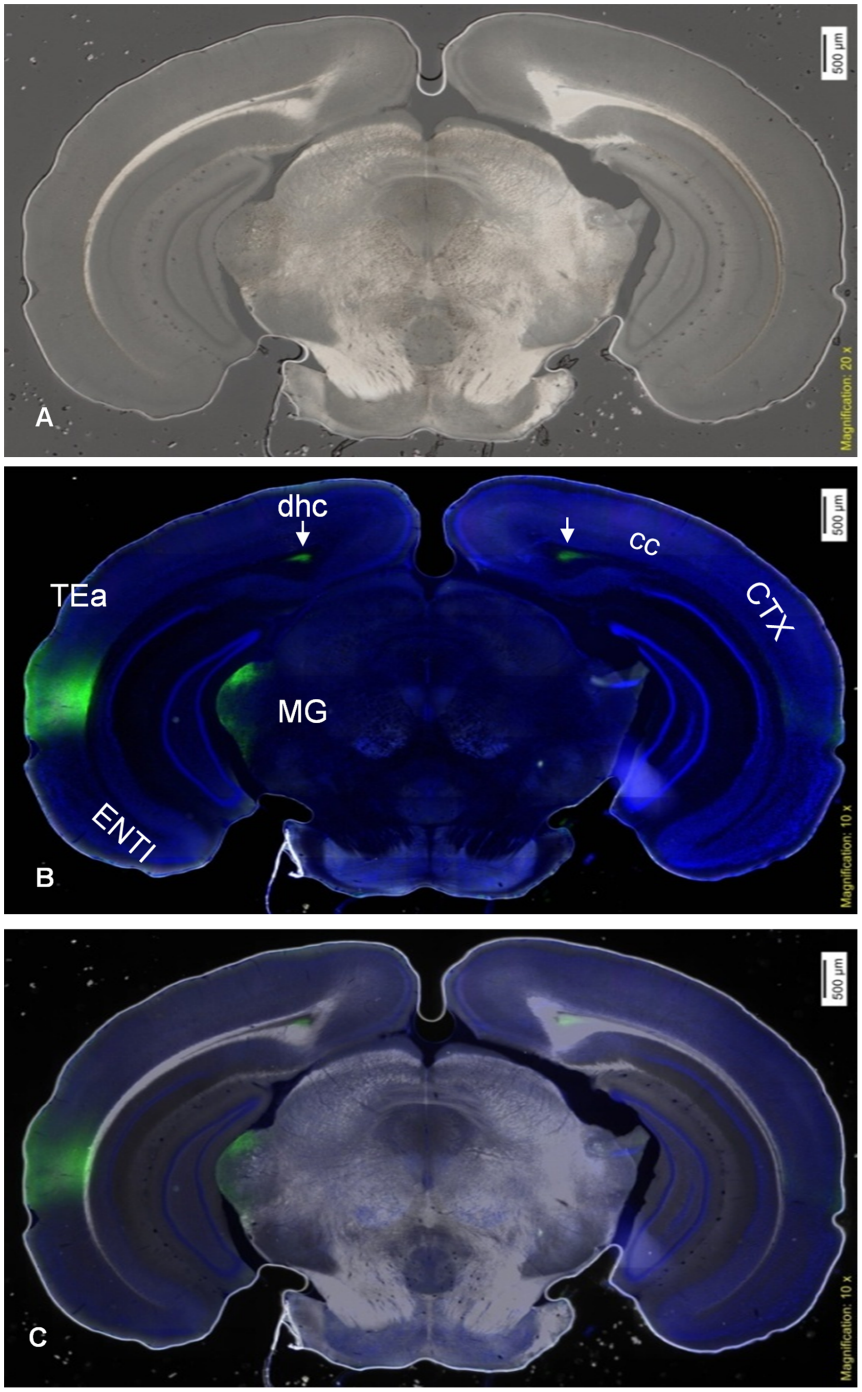
Images of *Phaseolus vulgaris* leucoagglutinin (PHAL)-labeled neuronal pathways (green areas). (8A) Darkfield DIRI image collected using a 20× objective lens. (8B) Fluorescence of PHAL-labeled neuronal pathways in green with DAPI stained features in Blue (shown in figure 8B) collected using a 10× objective lens and. (8C) A composite image of Figures 8A and 8B providing improved clarity of localization of PHAL-labeled neuronal pathways. (i.e., medial geniculate complex, MG) and white matter (i.e., dorsal hippocampal commissure, dhc). DIRI also enhances the appearance of white matter through which PHAL-labeled axons travel (indicated by arrows). Abbrevations include cc: corpus callosum; CTX: cerebral cortex; dhc: dorsal hippocampal commissure; ENTl: lateral part of entorhinal cortex; MG: medial geniculate complex; TEa: temporal association areas.

We imaged fluorescent molecules within the section of brain tissue using an incident light fluorescence illuminator. Fluorescent images were captured using a 10× objective lens (Figure 7A) and DIRI images were collected using a 20× objective lens. The images were then superimposed (Figure 7B). The composite image shows not only protein localization (green) but also details of myelinated tracts, and prominent cell groups, which facilitate identification of brain structures containing fluorescent label. Observation of DIRI (Figure 8A) and fluorescence (Figure 8B) channels, along with composite images of both techniques (Figure 8C) revealed the structure of the brain as well as protein distribution.

### 3. Conclusion: Overall Impression of DIRI

The extension of WSI to include darkfield imaging has great potential to expand the prospects for observation of brain tissue using light microscopes with a minimum of staining. Future development of side illumination with brighter LEDs, combined with more sensitive detectors, should provide greatly reduced exposure times and improved S:N ratios.

## Discussion

### 1. Expansion of the Applications

Our testing was limited to brain samples, but this WSI system may have utility beyond neuroscience research. We believe a WSI system may be helpful for observing a wide variety of cell and organ types, and indeed for observing almost any microscope slide sample that must be observed at high resolution. Additional applications include cancer research, urology and translational research, among numerous other fields.

### 2. Limitations

There are both advantages and disadvantages in using transmitted darkfield versus side-illumination darkfield. The transmitted darkfield illuminator provides high contrast and very bright darkfield images. But, the image produces a halo with a very bright edge surrounding the cell image. Furthermore, the substage condenser is limited in its NA and must be changed every time the objective is changed. Darkfield condensers used with objectives that have NAs higher than 0.9 require oil immersion condenser. In comparison, side-illumination darkfield works well with a wide range of objectives with markedly varying NAs.

Furthermore, dirt on the top or bottom of the slide may cause substantial light scattering and disrupt the quality of the image when using a traditional darkfield condenser; side-illumination darkfield is less sensitive to small particles that may settle on the slide or cover glass.

Exposure time is always an issue for WSI. The brightness of the illumination depends on the intensity of the LED and the availability of brighter LEDs will further reduce total acquisition time for DIRI systems.

#### 2-1. Illumination field

Using the described setup, side-illuminated darkfield works well at 10×, 20× and 40× magnifications and may work with other higher-magnification objective lenses. However, with lower-magnification lenses such as the 2× objective lens, LED illumination light reflects the edge of the slide glass and scatters light. This scattered light gets into the objective lens and may produce so-called image ghosting. This edge reflection also can reduce image contrast in cases where the specimen is close to the edge of the slide.

#### 2-2. Brightness of scattering light

Darkfield uses scattered light from both tissues/cells and the mounting medium to help visualize structure. The light-scatter intensity is dependent on the refractive index of the mounting medium and cell-tissue fixation protocol. If the refractive index difference is small, then the intensity of scattered light will be decreased, thus increasing exposure times.

#### 2-3. Color image acquisition

Collecting color images requires careful calibration of white balance. Traditional substage condensers typically use halogen light with a broad spectral output. Side-illuminated darkfield using the LED device that is described herein provides a more limited spectral output. The spectral output of LEDs should be selected with attention to the spectral properties of DAB reaction product, which is largely in the yellow-to-red color range.
